# Long-Term Stabilization Effects of Leptin on Brain Functions in a Leptin-Deficient Patient

**DOI:** 10.1371/journal.pone.0065893

**Published:** 2013-06-14

**Authors:** Sabine Frank, Martin Heni, Anja Moss, Julia von Schnurbein, Sadaf Farooqi, Hans-Ulrich Häring, Andreas Fritsche, Hubert Preissl, Martin Wabitsch

**Affiliations:** 1 Institute of Medical Psychology and Behavioral Neurobiology, fMEG Center, University of Tübingen, Tübingen, Germany; 2 Department of Internal Medicine IV, University Hospital, Tübingen, Germany; 3 Institute for Diabetes Research and Metabolic Diseases of the Helmholtz Center Munich at the University of Tübingen, Tübingen, Germany; 4 German Center for Diabetes Research, Neuherberg, Germany; 5 Division of Pediatric Endocrinology and Diabetes, Department of Pediatrics and Adolescent Medicine, University of Ulm, Ulm, Germany; 6 Metabolic Research Laboratories, Institute of Metabolic Science, Addenbrooke’s Hospital, University of Cambridge, Cambridge, United Kingdom; University of Las Palmas de Gran Canaria, Spain

## Abstract

**Context:**

Congenital leptin deficiency, caused by a very rare mutation in the gene encoding leptin, leads to severe obesity, hyperphagia and impaired satiety. The only systemic treatment is the substitution with metreleptin leading to weight reduction based on hormonal changes. Several studies have also shown alterations in brain function after metreleptin therapy. In a previous study, we were able to show changes in homeostatic (hypothalamus) and reward-related brain areas (striatum, orbitofrontal cortex (OFC), substantia nigra/ventral tegmental area, amygdala) 3 days and 6 months after therapy start in a leptin-deficient adolescent girl. To further access the time course of functional brain activation changes, we followed the patient for 2 years after initiation of the therapy.

**Design, Patient:**

Functional magnetic resonance imaging during visual stimulation with food (high- and low-caloric) and non-food pictures was performed 1 and 2 years after therapy start in the previously described patient.

**Results:**

The comparison of ‘food vs. non-food’ pictures showed a stabilization of the long-term effects in the amygdala and in the OFC. Therefore, no significant differences were observed between 6 months compared to 12 and 24 months in these regions. Additionally, a reduction of the frontopolar cortex activity over the whole time span was observed. For the comparison of high- and low-caloric pictures, long-term effects in the hypothalamus showed an assimilating pattern for the response to the food categories whereas only acute effects after 3 months were observed in hedonic brain regions.

**Conclusion:**

This follow-up study shows that the long lasting benefit of metreleptin therapy is also associated with activation changes in homeostatic, hedonic and frontal control regions in congenital leptin deficiency.

## Introduction

Leptin plays a major role in energy homeostasis mainly by its effect in the hypothalamus [1]. Hardly detectable leptin levels are the result of congenital leptin deficiency, a very rare genetic malfunction [2]. Leptin-deficient patients are severely obese and suffer from hyperphagia, impaired satiety, immunological differences and abnormal pubertal development [3]. After leptin replacement therapy with recombinant human metreleptin, patients show dramatic weight reduction and normalization of associated symptoms [3]–[7].

Few neuroimaging studies provide insight in differential brain functions which are due to leptin deficiency and metreleptin therapy [8]–[11]. Those studies mainly showed differences in gustatory (insular cortex, orbitofrontal cortex (OFC)), reward (substantia nigra/ventral tegmental area (SN/VTA), amygdala, striatum), and homeostatic areas (hypothalamus) but also in frontal control regions and in the cerebellum. In such studies, acute effects (several days) and long-term effects after several months were described.

In our previous study [10], we observed acute (3 days) and long-term effects (6 months) on brain activation after visual stimulation with food (high- and low-caloric) and non-food cues measured by functional magnetic resonance imaging (fMRI). Activation differences between ‘food vs. non-food’ stimulation revealed decreased amygdala activation and in the SN/VTA over time and an increased activation in the OFC. Acute effects after three days were observed in food reward regions (striatum, OFC) after stimulation with high- vs. low-caloric pictures. Comparing brain responses to high- versus low-caloric pictures, we also observed a long-term alteration in the hypothalamus, the major homeostatic control center. Here, the activation to high-caloric pictures decreased over time, while low-caloric stimuli led to increased activation. Since congenital leptin deficiency is so rare, we are especially interested in the stability of the findings in this patient obtained after therapy start. Furthermore, it is of great interest if leptin replacement causes further changes of the associated brain pattern in the long run. Thus, we report the 1- and 2-years follow-up study of a leptin-deficient Austrian girl including also the first three measurements (pre, 3 days, and 6 months). We hypothesize maintenance of the long-term effects and stabilization of the previously found brain pattern.

## Methods

### Case History

Previously, we reported the case of a patient carrying a homozygous mutation in the LEP gene [12]. Starting at the age of 14, the patient was supplemented with human metreleptin (0.6 mg twice daily; Amylin Pharmaceuticals, Inc), which led to a dramatic reduction of her BMI from 36 kg/m^2^ to 27 kg/m^2^ followed by a stabilization after 1 year (Table 1), and also to endocrine changes including the onset of menstrual cycle [6]. Alterations in brain function were reported up to 6 months after the onset of the substitution therapy [10]. The study was approved by the ethics committee of the University of Ulm. Both parents of the patient as well as the patient herself provided written informed consent.

**Table 1:** Questionnaire and patient characteristics.

	Pre	3 days	6 months	12 months	24 months	Scale range
BMI (kg/m^2^)	35.9	35.9	29.7	26.9	27.5	
Fat mass (%), DEXA	50.1	n.d.	40.2	37.8	38.0	
TFEQ: Cognitive control	21	21	21	20	21	0–21
TFEQ: Disinhibition	3	4	3	2	3	0–16
TFEQ: Experienced hunger	1	2	0	0	0	0–14
BDI	1	0	2	0	0	0–63

BMI, Body Mass Index; BDI, Beck Depression Inventory; TFEQ, Three Factor Eating Questionnaire; n.d. not determined.

### Experimental Design and Procedure

The protocol of this study conforms to the protocol of the initial study [10], in which we conducted fMRI measurements at 3 visits: 6 days pre metreleptin substitution, 3 days and 6 months after beginning of the therapy. In order to evaluate a long term follow-up, the same fMRI protocol was performed 1 and 2 years after start of the therapy. All measurements were performed at the same time of day at 11 am after an overnight fast. Additionally, body fat percentage was assessed by dual-energy x-ray absorptiometry.

### Behavioral Data

At each measurement day the German version of the Three Factor Eating Questionnaire (TFEQ) [13] and the Beck Depression Inventory (BDI) [14] were applied. Additionally, the subject rated high- and low-caloric food pictures for palatability on a 5-point Likert scale each day. Here, the patient was asked how palatable she evaluated each picture which was previously shown in the scanner. This rating represents the Liking component of the food-reward construct [15]. Behavioral data were collected after the scanning session and statistically analyzed with SPSS 18 (SPSS Inc, Chicago, IL) using ANOVA with P<0.05 and consecutive post-hoc tests with P<0.05 Bonferroni corrected. Additionally, Spearman’s Rho correlation analyses were performed in order to examine the association of palatability ratings and the fMRI results.

### Imaging Procedures

Whole-brain fMRI blood oxygen level dependent (BOLD) data were obtained in a 3.0T scanner (Siemens Trio, Erlangen, Germany). Each of three sessions consisted of 226 scans (TR = 2 s, TE = 30 ms, matrix 64×64, flip angle 90°, voxel size 3×3×3 mm^3^, slice thickness 3 mm, 0.75 mm gap, 30 slices, images acquired in ascending order). During the fMRI scanning the patient was stimulated with food (F), divided into high-caloric (HC) and low-caloric (LC), and non-food (NF) pictures. Visual stimulation was performed by using Presentation® software (Version 10.2, www.neurobs.com) and a custom-made visual stimulation device (mirror) to project the visual cues into the patient’s field of view. The pictures of the different categories were matched for complexity, valence and arousal and were presented in a block design within a one-back task. Here, the patient had to press a button as fast as possible to indicate whether the shown picture was the same as the picture immediately shown before (left button) or not (right button). Details on the stimulus material and experimental paradigm have previously been described [16].

Additionally, high-resolution T1 weighted anatomical images were obtained.

### Imaging Analysis

Analysis of the fMRI data was performed with SPM8 (http://www.fil.ion.ucl.ac.uk/spm/). Standard preprocessing including realignment, coregistration to the anatomical T1 weighted image, normalization into MNI space (3 mm isotropic voxel size) and Gaussian spatial smoothing (FWHM: 6 mm) was performed. Data were high-pass (cut off: 128 s) and autocorrelation corrected (AR(1)). In the fixed effect analysis for each condition a separate regressor was modeled using a canonical hemodynamic response function (HRF) including time derivatives. Movement parameters were modeled as confounds. A fixed effect analysis was applied using the following factors: *food* (F vs. NF) and *calorie content* (HC vs. LC). Based on the results of our previous study, interactions of the factors *food* and *calorie content* with the factor *time* were analyzed with a region-of-interest (ROI) approach for the regions which had shown significant changes in the previous study: hypothalamus, ventral striatum, SN/VTA, amygdala, OFC. To explore further effects, a whole-brain analysis was performed in addition. Results were considered significant with *P*_FWE_<0.05, family wise error corrected.

## Results

### Behavioral Data

**Figure 1:**
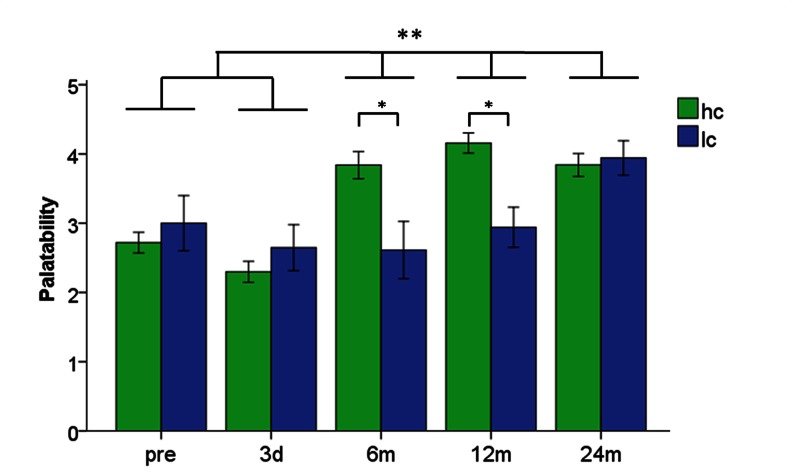
Palatability rating of high-caloric (hc) and low-caloric (lc) food pictures. The Y-axes shows the palatability score (range: 1–5) ± SEM. * Palatability ratings for high- and low-caloric food pictures were significantly different to 6 and 12 months after therapy start at *P*<0.005 Bonferroni corrected **time points pre and 3 days showed significantly lower palatability ratings compared to 6, 12 and 24 months at *P*<0.005 Bonferroni corrected.

The subject showed no depressive symptoms but high cognitive control, low disinhibition, and very low reported hunger at each measurement day (Table 1). The palatability ratings of high- and low-caloric food, however, changed over the two years (main effect of time: F_(4,359)_ = 10.05, P<0.001). Also the main effect *food* (HC vs. LC) reached significance indicating higher overall palatability rating for HC food items (F_(1,359)_ = 4.76, P = 0.03). This effect is driven by significantly higher scores for the high-caloric food after 6 and 12 months compared to low-caloric food (both P<0.005). However, since the palatability ratings of low-caloric food increased over the two years, an assimilation of high- and low-caloric stimuli occurred after 24 months (Figure 1).

### Imaging Data

#### Food x Time

**Figure 2:**
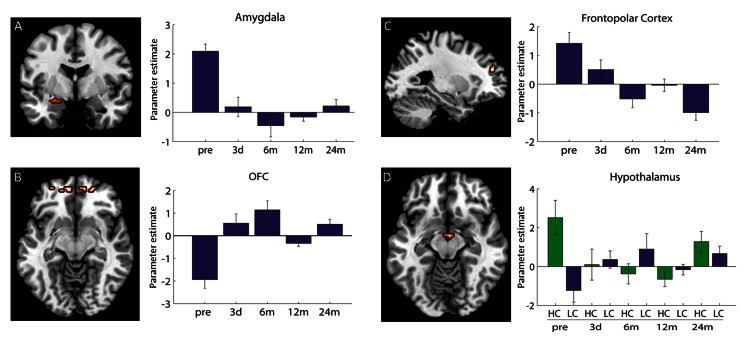
Imaging data. A left: Coronal view of amygdala activation for interaction food (F) vs. non-food (NF) over time. B: left: Transversal view of OFC activation for interaction F vs. NF over time. C left: Sagittal view of the frontopolar activation for interaction F vs. NF over time. A-C right: Activation difference of F-NF pictures at five measurement times. D left: Transversal view of the hypothalamic activation for the interaction of high- vs. low-caloric (HC vs. LC) stimuli over time. D: right: activation difference of HC and LC displayed separately for both calorie contents. The bar-plots represent parameter estimates ± SEM. (A: y = −3, B: x = −27, C: z = −9, D: z = −12).

The contrast ‘food vs. non-food’ revealed significant long-term response differences in the left amygdala over time. After the decrease in activity during the first six months, the follow-up measurements showed no further difference (Figure 2A, Table 2). The same pattern was found in the SN/VTA, however, this effect was not significant with the applied statistical threshold (Figure S1). Also the OFC showed significant differences after 24 months in comparison to the leptin-deficient state (pre) (Figure 2B, Table 2). In addition to the ROI analyses, a whole-brain analysis was performed, showing long-term changes up to 24 months for frontopolar regions (Figure 2C, Table 2). Correlation analysis of the frontopolar cortex with the palatability rating revealed a negative Spearman’s Rho correlation of r = −0.8 which was significant at the trend level (p = 0.10). No significant change in the activation pattern for any of these regions from 6 months to 12 and 24 months was observed (Table 2).

**Table 2:** Brain Imaging results.

Contrast	Brain Region	Coordinates			K	F value	F value		F value		F value		
		x	y	z		(pre vs. 12 m)		(pre vs. 24 m)		(6 m vs. 12 m)		(6 m vs. 24 m)	
F - NF	Amygdala	−24	−3	−15	46	20.17		19.17		n.s.		n.s.	
F - NF	OFC	−12	54	−15	23	n.s.		28.19		n.s.		n.s.	
		21	54	−9	80	n.s.		25.02		n.s.		n.s.	
HC - LC	Hypothalamus	3	−6	−12	7	18.12		n.s.		n.s.		n.s.	
F - NF*	Frontopolar cortex	−27	48	30	18	13.50		39.68		n.s.		n.s.	

Contrast, Brain region, MNI coordinates, cluster size k, F-values. All data are significant at p(FWE)<.05 family wise error corrected.

* result of the whole-brain analyses without ROI mask.

#### Calorie content x time

The long-term effect up to 24 months after therapy start in the hypothalamus consists in an assimilating pattern of the response to high- and low-caloric pictures (Figure 2D, Table 2). No significant changes in the activation pattern for the hypothalamus from 6 months to 12 and 24 months were observed. Analyses of the first three measurements revealed acute effects in the ventral striatum and the OFC (after 3 days). When including the follow-up measurements, no significant effect in these regions were observed (Figure S2, S3). No further effect in the whole-brain analysis was observed.

## Discussion

### Behavioral Data

In the leptin-deficient state, the patient stands out with regard to her very high cognitive control which presumably is due to the need to keep the low-caloric diet her whole life. This very high cognitive control level was stable up to 24 months after therapy start even though she had been allowed to eat less restricted for two years. Additionally, changes in the palatability ratings of food cues were observed. Those changes demonstrate alterations in her food preference after therapy start, with higher palatability ratings for high-caloric food and for food in general.

### Imaging Data

#### Food x time

A stabilized pattern was observed for the amygdala and the OFC in the contrast ‘food vs. non-food’. The response in these areas seems to plateau which is also represented in non-significances in the contrasts between 6 months to 12 months and 24 months. We interpret the decreased amygdalar activity as a change in the emotional salience of food in comparison to non-food stimuli. The amygdala’s role in emotional processing also includes the detection of salient and individually relevant stimuli [17]. After therapy start, the patient was released from the strict low-caloric diet and the emotional salience of food items may have been attenuated. This effect may be represented in decreased amygdala activation due to food pictures in comparison with non-food items. In addition, the amygdala also receives projections from the hypothalamus [18]. Hypothalamic activity is strongly affected by leptin and weight-loss and the reduced amygdala activation might also be influenced by weight-loss [19].

Another main component of the processing of food pictures, besides the emotional component, is cognitive control which is particularly high in this patient. On a neural level, cognitive control is associated with prefrontal cortex activity which is integrated in the cognitive control circuitry [20]. Here, we found a continuous decrease of frontopolar activity, suggesting a decrease in cognitive control in association with food after therapy start and the release from the low-caloric diet. This is particularly interesting since questionnaire data suggest high stable cognitive control pattern. However, the subjective ratings of palatability increased and showed a rather high correlation with the BOLD response in the frontopolar cortex. Despite the fact, that this correlation coefficient was rather high, the correlation did not reach significance with the used threshold. This was probably due to the small sample size of only five measurements. This result is conclusive as regards the patient’s release from her strict diet and the ability to enjoy food differently without the permanent need for central control mechanisms. Subjects losing weight by conventional methods (sports, diet), show heightened activity in frontal control regions after losing weight and keep the weight loss successfully. Those results are interpreted as the input of inhibitory neural systems of the frontal cortex, based on the high effort of self-control to keep the weight loss [21]. On the other hand, the reduced frontopolar cortex activity found in our study is in line with studies assessing functional brain pattern after bariatric surgery. Similar to the situation of this leptin-deficient patient, bariatric surgery leads to significant weight loss-based changes in the physiology (here reduced stomach size). Therefore, also in bariatric patients weight reduction occurs without the need for excessive inhibitory control and is associated with reduced prefrontal cortex activity after surgery [22], [23]. Thus, we assumed that a reduction in the frontopolar cortex activity would go along with a reduction in the cognitive control score according to the TFEQ. However, the patient still showed high cognitive control scores 2 years after therapy start. This may be based on the high impact of learned habits and very strict behavioral manners she acquired. This is supported by the finding that in other leptin-deficient patients, who do not show such high cognitive control, a pause of substitution therapy led to lower prefrontal cortex activity and weight gain [9]. We suggest that the reduced frontopolar activity combined with unchanged high scores of cognitive control represents decreased neuronal effort for the patient to maintain such high cognitive control. The cognitive control pattern, therefore, might be the key mechanisms of the processing of food stimuli in this patient.

#### Calorie content x time

Interestingly, the homeostatic and reward-related areas show completely different activation pattern after stimulation with high- and low-caloric food items. While the hypothalamus as the homeostatic control center reveals long-term adaptations, hedonic regions showed only acute effects after 3 days. Higher brain functions like reward and cognitive functions are under the influence of metabolic signals in a bottom-up process [24]. In our subject this bottom-up signal was missing before treatment and the subject developed an even stronger psychological component to control eating behavior also by weakening the rewarding value of high- and low-caloric food. After therapy start, the bottom-up signal was introduced and functional, which may have affected reward and cognitive systems immediately. In contrast, the homeostatic system shows bottom-up and top-down processes. Due to her high cognitive control the patient was able to override the bottom-up signal by top-down modulation.

In summary, this follow-up study shows a stabilization of the brain pattern 24 months after the start of metreleptin substitution. None of the regions influenced by the substitution therapy showed significant differences between 6 to 12 months and 24 months after the start of substitution. Interestingly, the hedonic system shows rather acute effects whereas the homeostatic system reveals long-term adaptive changes which suggest different underlying processes. This study is the first to show food-related neurophysiological effects in repeated fMRI measurements before and up to 2 years after initiation of metreleptin substitution in a leptin-deficient patient.

## Supporting Information

Figure S1 **Long term effect in the substantia nigra/ventral tegmental area (SN/VTA) for the contrast ‘food vs. non-food’.** Left: Coronal view of the SN/VTA for the interaction high- vs. low-caloric (HC vs. LC) stimuli over time; activation differences significant only when first three measurements are considered (pre, 3 days, 6 months, see Frank et al., 2011). Right: Activation difference of HC vs. LC pictures at five measurement times. The bar-plots represent parameter estimates ± SEM.
(TIF)

Figure S2 **Acute effect in the striatum for the interaction ‘calorie content x time’.** Left: Coronal view of the ventral striatum for the interaction high- vs. low-caloric (HC vs. LC) stimuli over time. Right: Activation difference of HC vs. LC pictures at five measurement times. The bar-plots represent parameter estimates ± SEM.
(TIF)

Figure S3 **Acute effect in the orbitofrontal cortex (OFC) for the interaction ‘calorie content x time’.** Left: Coronal view of the OFC for the interaction high- vs. low-caloric (HC vs. LC) stimuli over time. Right: Activation difference of HC vs. LC pictures at five measurement times. The bar-plots represent parameter estimates ± SEM.
(TIF)
