# Supplementary figures and images for: Long-Term Stabilization Effects of Leptin on Brain Functions in a Leptin-Deficient Patient
Source: PLoS One. 2013 Jun 14;8(6):e65893. doi: 10.1371/journal.pone.0065893 (PMC3683048; doi:10.1371/journal.pone.0065893)

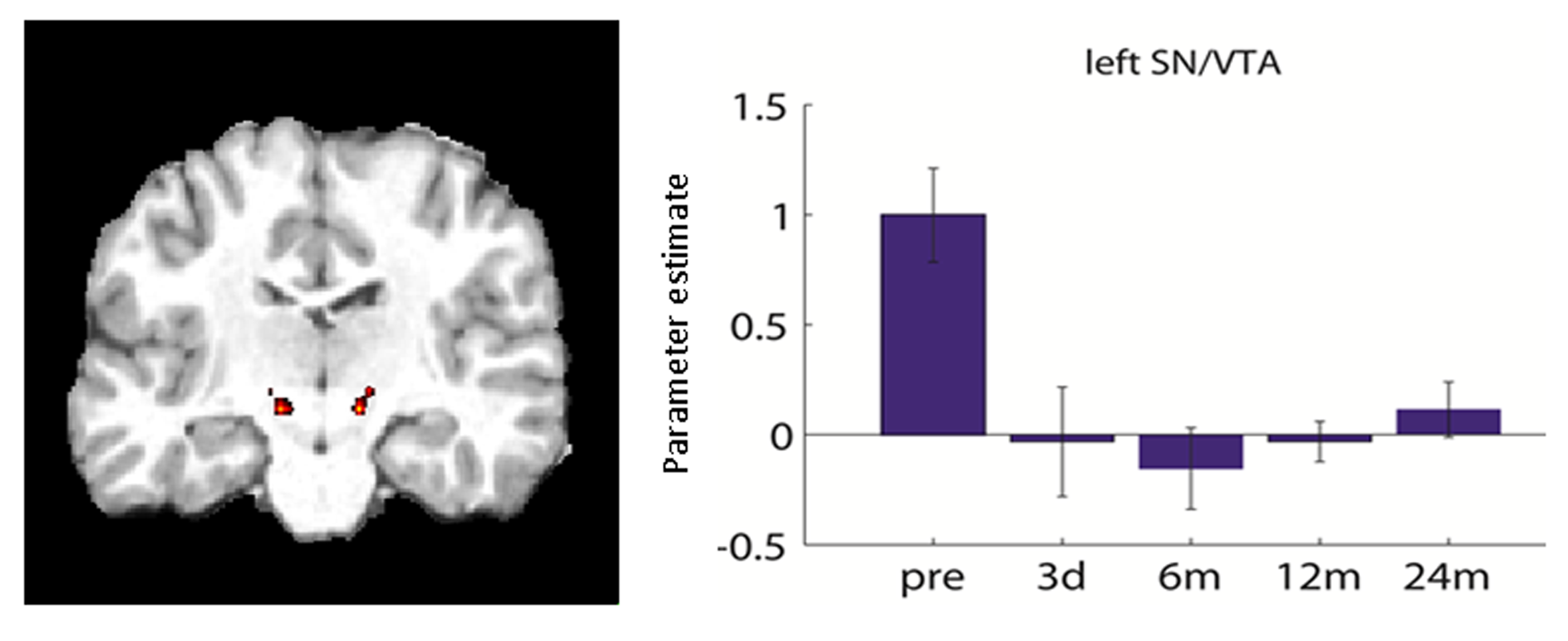

Supplement: Figure S1 — Long term effect in the substantia nigra/ventral tegmental area (SN/VTA) for the contrast ‘food vs. non-food’. Left: Coronal view of the SN/VTA for the interaction high- vs. low-caloric (HC vs. LC) stimuli over time; activation differences significant only when first three measurements are considered (pre, 3 days, 6 months, see Frank et al., 2011). Right: Activation difference of HC vs. LC pictures at five measurement times. The bar-plots represent parameter estimates ± SEM. (TIF) [file pone.0065893.s001.tif]

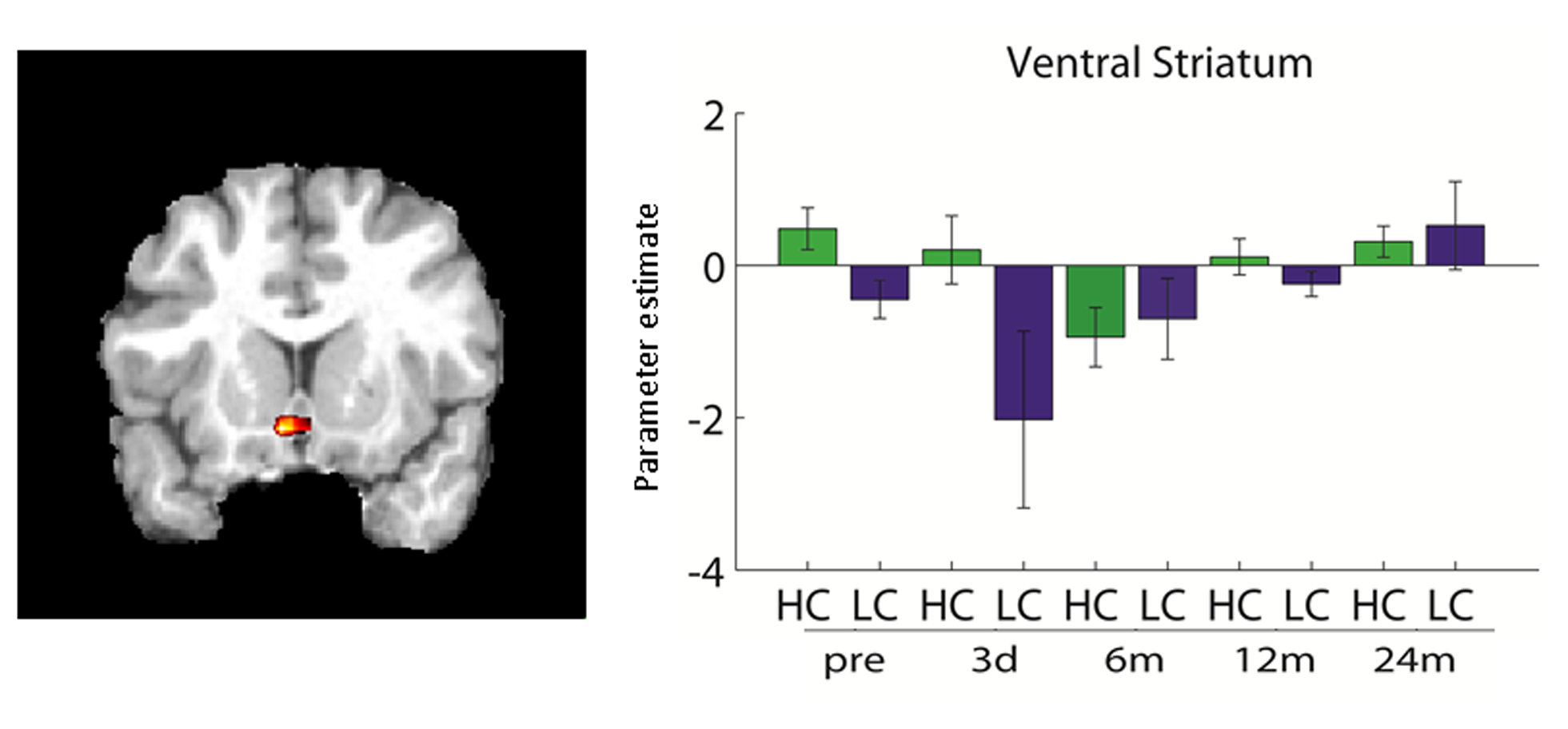

Supplement: Figure S2 — Acute effect in the striatum for the interaction ‘calorie content x time’. Left: Coronal view of the ventral striatum for the interaction high- vs. low-caloric (HC vs. LC) stimuli over time. Right: Activation difference of HC vs. LC pictures at five measurement times. The bar-plots represent parameter estimates ± SEM. (TIF) [file pone.0065893.s002.tif]

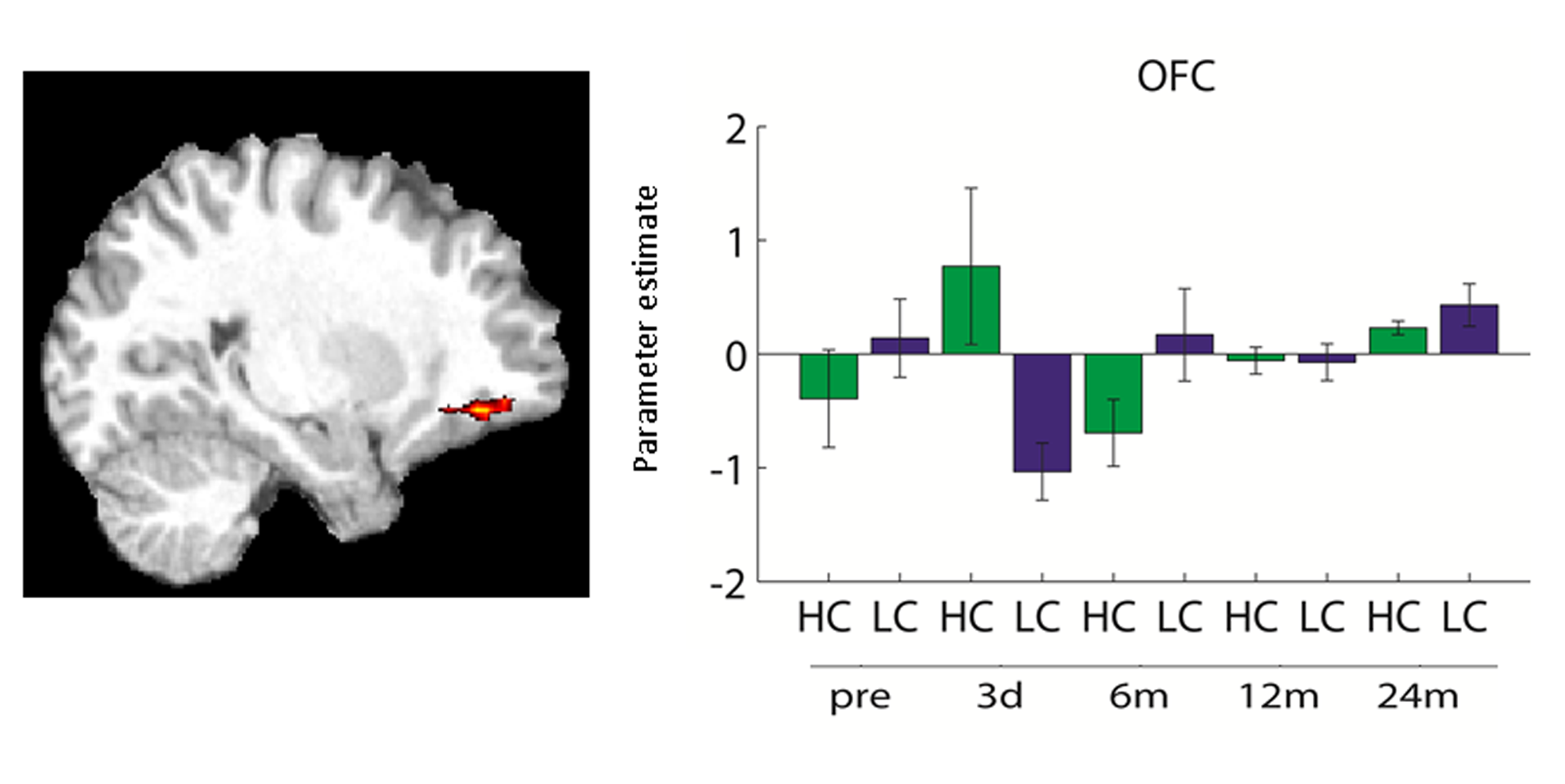

Supplement: Figure S3 — Acute effect in the orbitofrontal cortex (OFC) for the interaction ‘calorie content x time’. Left: Coronal view of the OFC for the interaction high- vs. low-caloric (HC vs. LC) stimuli over time. Right: Activation difference of HC vs. LC pictures at five measurement times. The bar-plots represent parameter estimates ± SEM. (TIF) [file pone.0065893.s003.tif]
